# Society Meetings

**Published:** 1906-03

**Authors:** 


### SOCIETY MEETINGS.

THE American Medical Association will hold its next annual meeting at Boston, June 5-8, 1906, under the following administration:

PRESIDENT—Lewis S. McMurtry, Louisville, Ky.
PRESIDENT-ELECT—William J. Mayo, Rochester, Minn.
FIRST VICE-PRESIDENT—Walter Wyman, Washington, D. C.
SECOND VICE-PRESIDENT—K. A. J. Mackenzie, Portland, Oregon.
THIRD VICE-PRESIDENT—Eugene S. Talbot, Chicago.
FOURTH VICE-PRESIDENT—E. D. Martin, New Orleans.
GENERAL SECRETARY AND EDITOR—George H. Simmons, 103 Dearborn Ave., Chicago.
TREASURER—Frank Billings, Chicago.
CHAIRMAN COMMITTEE OF ARRANGEMENTS—Herbert L. Burrell, 22 Newbury St., Boston.
BOARD OF TRUSTEES—William H. Welch, Baltimore, 1906; Miles F. Porter, Ft. Wayne, Ind., 1906; M. L. Harris, Secretary, Chicago, 1906; T. J. Happel, Chairman, Trenton, Tenn., 1907; W. W. Grant, Denver, Colo., 1907; Philip Marvel, Atlantic City, N. J., 1907; E. E. Montgomery, Vice Chairman, Philadelphia, 1908; A. L. Wright, Carroll, Iowa, 1908; H. L. E. Johnson, Washington, D. C., 1908
JUDICIAL COUNCIL—P. Maxwell Foshay, Chicago, Chairman; D. C. Peyton, Jeffersonville, Ind.; George Ben Johnston, Richmond, Va.; W. B. Russ, San Antonio, Texas; W. S. Foster, Pittsburg, Pa.
COUNCIL ON MEDICAL EDUCATION—Arthur D. Bevan, Chicago, Chairman; W. T. Councilman, Boston; J. A. Witherspoon, Nashville, Tenn.; Charles H. Frazier, Philadelphia; Victor C. Vaughan, Ann Arbor, Mich.
COUNCIL ON PHARMACY AND CHEMISTRY—G. H. Simmons, Chairman, Chicago; C. S. N. Halberg, Secretary, Chicago; A. R. Cushny, Ann Arbor, Mich.; C. Lewis Diehl, Louisville, Ky.; R. A. Hatcher, New York City; L. F. Kebler, Washington, D. C.; J. H. Long, Chicago; F. G. Novy, Ann Arbor, Mich.; W. A. Puckner, Chicago; S. P. Sadtler, Philadelphia; J. O. Schlotterbeck, Ann Arbor, Mich.; Torald Sollmann, Cleveland; Julius Stieglitz, Chicago; M. I. Wilbert, Philadelphia; H. W. Wiley, Washington, D. C.

Orator on Medicine—F. C. Shattuck, Boston.
Orator on Surgery—Joseph D. Bryant, New York.
Orator on State Medicine—W. H. Sanders, Montgomery, Ala.

---

The Homeopathic Medical Society of the State of New York at its recent annual meeting, held at Albany, elected officers for the current year as follows: president, Newton W. Collins, Rochester; first vice-president, T. Drysdale Buchanan, New York; second vice-president, Frederick W. Seward, Jr., Goshen; third vice-president, O. S. Ritch, Brooklyn; secretary, H. Worthington Paige, Oneonta; treasurer, F. M. Dearborn, New York; necrologist, John L. Moffat, Brooklyn; counsel, P. E. Wadhams, Albany.

---

The Buffalo Academy of Medicine held meetings during the month of February as follows:

*Section of Surgery.*—Tuesday, February 6, 1906, at 8:30 P. M. Program: (a) Tuberculosis and tumors of the kidney, Arthur Dean Bevan, Chicago. (b) Review of the literature of morphine-scopolamine anesthesia, Lawrence Hendee.

*Section of Pathology.*—Tuesday, February 13, 1906. at 8:30 P. M. Program: Clinical examination of feces, Richard Weil, New York City; discussion opened by Dr. A. E. Woehnert.

*Section of Medicine.*—Tuesday, February 20, 1906, at 8:30 P. M. Program: (a) Longevity among Yale Athletes, William Gilbert Anderson, M.A., M.D., Director Yale University Gymnasium, New Haven, Conn. (b) Functional derangement of the heart, James S. Smith.

*Section of Obstetrics and Gynecology.*—Tuesday, February, 27, 1906, at 8:30 P. M. Program: The limitations of some of the common gynecological procedures, Hunter Robb, Cleveland, Ohio.

---

The American Gastroenterological Association will hold its ninth annual meeting at Boston, June 4 and 5, 1906, under the presidency of Dr. H. W. Bettmann, of Cincinnati. This society has already issued its preliminary program, containing fifteen titles of papers.

